# Human Peripheral Nerve-Derived Pluripotent Cells Can Be Stimulated by In Vitro Bone Morphogenetic Protein-2

**DOI:** 10.3390/bioengineering8100132

**Published:** 2021-09-26

**Authors:** Renyi Sun, Tanghong Jia, Bradley Dart, Sunaina Shrestha, Morgan Bretches, Michael H. Heggeness, Shang-You Yang

**Affiliations:** 1 Jinan Central Hospital Affiliated to Shandong University, Jinan 250013, China; renyisun@sdu.edu.cn (R.S.); z18503603@163.com (T.J.); 2 Department of Orthopaedic Surgery, University of Kansas School of Medicine-Wichita, Wichita, KS 67214, USA; bdart@kumc.edu (B.D.); heggeness@msn.com (M.H.H.); 3 Department of Biological Sciences, Wichita State University, Wichita, KS 67260, USA; sunainashrestha1987@gmail.com (S.S.); mpbretches@shockers.wichita.edu (M.B.)

**Keywords:** pluripotent stem cells, BMP-2, peripheral nerve, human

## Abstract

We have recently identified a population of cells within the peripheral nerves of adult rodent animals (mice and rats) that can respond to Bone Morphogenetic Protein-2 (BMP-2) exposure or physical injury to rapidly proliferate. More importantly, these cells exhibited embryonic differentiation potentials that could be induced into osteoblastic and endothelial cells in vitro. The current study examined human nerve specimens to compare and characterize the cells after BMP-2 stimulation. Fresh pieces of human nerve tissue were minced and treated with either BMP-2 (750 ng/mL) or a PBS vehicle for 12 h at 37 °C, before being digested in 0.2% collagenase and 0.05% trypsin-EDTA. Isolated cells were cultured in a restrictive stem cell medium. Significantly more cells were obtained from the nerve pieces with the BMP-2 treatment in comparison with the PBS vehicle controls. Cell colonies started to form at Day 3. Expressions of the four transcription factors, namely, Klf4, c-Myc, Sox2, and Oct4, were confirmed at both the transcriptional and translational levels. The cells can be maintained in the stem cell culture medium for at least 6 weeks without changing their morphology. When the cells were transferred to a fibroblast growth medium, dispersed spindle-shaped motile cells were noted and became fibroblast activated protein-α (FAP) positive with immunocytochemistry staining. The data suggest that human peripheral nerve tissue also contains a population of cells that can respond to BMP-2 and express Klf4, Sox2, cMyc, and Oct4—the four transcription factors driving cell pluripotency. These cells are able to differentiate into FAP-positive fibroblasts. In summary, in human peripheral nerves also reside a population of quiescent cells with pluripotency potential that may be the same cells as rodent nerve-derived adult stem (NEDAPS) cells. It is proposed that these cells are possibly at the core of a previously unknown natural mechanism for healing an injury.

## 1. Introduction

The potential for stem cells to treat human disease is rightly perceived to be vast. Embryonic stem cells (ESCs) from the inner cell mass of mammalian blastocyst that have unlimited self-renewal and pluripotency can differentiate into ectodermal, mesodermal, and endodermal cells [1,2,3]. Although there are numerous ongoing studies to investigate the therapeutic potential of human embryonic stem cells (hESCs) for type I diabetes (T1D), heart failure, Parkinson’s disease, and inherited or acquired retinal degenerations [4], challenges remain to be conquered in the clinical development of hESCs, such as legal and ethical issues, immune rejections, and differentiation difficulties [3]. Somatic cells can be introduced to transform into a state of pluripotency [3]. The brilliant work of Drs. Yamanaka and Takayashi [5,6] demonstrated that pluripotent cells can be created from adult differentiated cells by the virally induced manipulation of nuclear genes to force expression of four specific transcription factors, namely, octamer-binding transcription factor 4 (Oct4), sex determining region Y-box 2 (Sox2), Krüppel-like family of transcription factor 4 (Klf4), and c-Myc, which together will convert the cells into pluripotency [7,8]. Induced pluripotent stem cells (iPSCs) have emerged as an intriguing and promising strategy in tissue regeneration and personalized medicine since the iPSCs can be harvested and processed from individual patient [9]. Unfortunately, this process of gene manipulation creates a very real risk of malignant transformation and may not solve the issue of immune rejection. Another drawback is that such cells are expensive to create and assessing the cells for risk of malignant transformation adds further time and expense [10,11,12]. 

We have serendipitously discovered a population of pluripotent cells that reside in a quiescent state within mouse peripheral nerves [13,14]. When the nerves are stimulated with a physical insult (including mechanical compression or stretching, exposure to blood, and electrical or cytokine BMP-2 stimulation), a massive proliferation of cells within the nerve results, with a rapid egress of the cells into the surrounding tissues. These proliferating cells uniformly exhibit expression of the four critical genes associated with pluripotency—Sox2, Oct4, c-Myc, and Klf4—as demonstrated by double-stain immunohistochemistry and by Real-Time Polymerase Chain Reaction (RT-PCR) with the appropriate primers [14]. They are readily cultured in restrictive serum-free media, adhere to substrate, and appear motile. They have been successfully differentiated into cells of the three primary germ layers: mesoderm (osteoblasts and endothelial cells) [13], endoderm (Definitive Endoderm), and ectoderm (Primitive Nerve Cells) in rodents (unpublished data). We have termed these cells Nerve-Derived Adult Pluripotent Stem cells, or NEDAPS cells. The discovery of this population of naturally existing pluripotency potential cells would address many currently debated issues, such as nerve dependence in tissue and organ regeneration [15], and may significantly change the concept of tissue-engineering therapies.

Indeed, there are too many instances where biological data from rodent experiments do not conform to human tissue responses. The objective of this study was to examine if human peripheral nerves also contain this population of quiescent pluripotent stem cells that can be stimulated by in vitro challenge of BMP-2. Cell differentiation potential was also principally tested.

## 2. Materials and Methods

### 2.1. Human Peripheral Nerve Tissue Treatment

This study was exempted by the Institutional Review Board (IRB) as a non-human investigation. Fresh human tibial nerves were obtained as surgical waste in a completely anonymous fashion from three serious trauma patients during limb amputation. These tissues would normally be discarded. No identifying features of the specimens were shared or recorded. The peripheral nerve tissues were stored in sterile normal saline on ice and transferred to the research lab within one hour from amputating the limb. The nerve specimens were rinsed twice in cold normal saline to remove blood and we carefully dissected any soft tissue debris attached, and then minced the samples to 0.5 cm pieces into 6-well culture plates (CytoOne, USA Scientific, Ocala, FL, USA) in DMEM medium, and 275 ng/mL of rhBMP-2 (InFuse™, Medtronic, Memphis, TN) was added to each well. For the controls, 100 µL of sterile saline (PBS) was added to the wells of the plate. The minced nerve tissue was incubated overnight at 37 °C before cell isolation.

### 2.2. Cell Isolation and Culture

Proliferating cells were isolated from the obtained human nerve specimens similar as reported previously for mouse NEDAPS cells [14]. Briefly, the minced nerve pieces were pelleted by centrifugation at 500× *g* for 5 min followed by digestion in 0.2% (0.27 U/mL) collagenase (Worthington Biochemical Corp, Lakewood, NJ, USA) at 37 °C for 90 min. An equal volume of 0.05% trypsin-EDTA solution was then added for 5 min with agitation. The enzyme digestion was stopped by addition of heat-deactivated Fetal Bovine Serum (FBS) and the mixture was filtered through a 100 μm-sized mesh before centrifugation at 500× *g* for 10 min to pellet cells. The cells were seeded into 6-well culture dishes, or 8-well chamber-slides in the restriction stem cell medium with 20% Knockout Serum Replacement (KSR, Gibco), 100 μM MEM non-essential amino-acid solution (Gibco), 1x GlutaMAX™-I (Gibco), 55 μM β-mercaptoethanol (Gibco), and 20 ng/mL human leukemia inhibitory factor (LIF, Gibco). The cells were cultured at 37 °C, in a 5% CO_2_ atmosphere. For fibroblast induction, the human NEDAPS cells that have been cultured in the stem cell medium for at least 7–14 days were switched to the fibroblast induction medium containing 20 ng/mL recombinant human basic Fibroblast Growth Factor (rhFGF-2), 50 µg/mL ascorbic acid, and 10% FBS in DMEM, at a cell density of 5 × 10^5^/well on a 6-well plate. 

### 2.3. Real-Time PCR (RT-PCR) Test

For RNA isolation, the cells were resuspended and lysed in TRIzol solution (Life Technologies, Carlsbad, CA, USA), and then went through chloroform separation and isopropanol precipitation following the protocol described by Chomczynski [16,17]. Complementary DNA (cDNA) was made by reverse transcription in the 40 μL mixture as follows: 4 μL 10 × PCR Rxn buffer (-MgCl_2_) (Invitrogen, Grand Island, NY, USA), 4.4 μL MgCl_2_ in the concentration of 5.5 mM (Invitrogen), 8 μL deoxynucleoside triphosphates in the concentration of 500 μM (Invitrogen), 2 μL RNase inhibitor in the concentration of 0.5 U/μL (Invitrogen), 2 μL random hexamers in the concentration of 2.5 μM (Invitrogen), 0.25 μL reverse transcriptase in the concentration of 1.25 U/μL (Invitrogen), 9.35 μL DNAse, RNAse-free water (Invitrogen), and 0.5 μg of the extracted RNA. The reactions were in fast reaction tubes on a Veriti 96-well Thermal Cycler (Applied Biosystems, Foster City, CA, USA). The temperature cycle was 25 °C for 10 min, 48 °C for 30 min, and 95 °C for 5 min. RT-PCR was run on a StepOnePlus RT-PCR System (Applied Biosystems) for 40 cycles (95 °C for 15 s, 60 °C for 1 min) in a fast optical 96-well reaction plate. The 25 μL reaction mixture was mixed by a 12.5 μL SYBR Green PCR Master Mix (Applied Biosystems), 6 μL cDNA, 5.5 μL DNAse, RNAse-free water (Invitrogen), and 400 nM tested gene primer pairs. The RT-PCR System can automatically record all the fluorescent signals dynamically. The values of threshold cycle (Ct) at which a statistically significant increase in reporter-dye signals (ΔRn) is first detected as an indication of the relative expression of the targeted genes, with the Ct > 38 as no expression. We employed the Primer3 program (http://bioinfo.ut.ee/primer3, 9/30/2015) to design the primer pairs for each target gene [18,19] and made them using Sigma-Genosys (Woodlands, TX, USA). The gene sequences are described in Table 1.

### 2.4. Immunocytochemistry Staining and Image Acquisition

The immunocytochemistry staining technique for the four stem cell markers (c-Myc, Sox2, Klf4, and Oct4) has been reported previously [13,14]. The following primary antibodies were used as pairs for double staining: goat anti-Sox2 (Santa Cruz), goat anti-Klf4 (R&D), rabbit anti-c-Myc (Abcam), goat and rabbit anti-Oct4 (Abcam), and rabbit anti-Myelin (Abcam). Cells were double stained with goat and rabbit first antibody pairs (Oct4 + c-MyC, Sox2 + Klf4, Klf4 + Oct4, Klf4 + c-MyC, Sox2 + Oct4, and Sox2 + c-MyC). For fibroblast differentiation, rabbit anti-FGF-2 (basic Fibroblast Growth Factor, Abcam) and rabbit anti-mouse FAB (Fibroblast Activation Protein, Abcam) were used. Following the washes, the diluted secondary antibody (1:200) in 1 × PBS with 1% BSA was added onto the cells for 1 h at room temperature in the dark. The secondary antibodies utilized were donkey anti-rabbit IgG conjugated with Alexa Fluor 594 (Life Technologies, red fluorescence emission) and donkey anti-goat IgG with Alexa 488 (green fluorescence emission). DAPI Fluoromount G (Southern Biotech, Birmingham, AL, USA) was used to mount the coverslip and to counterstain the cell nuclei. Double-stained fluorescent images of the cells were acquired under a TCS SP5 II confocal laser scanning microscope (Leica Microsystems). Acquisition of an automated sequential collection of multichannel sections was processed to reduce the spectral cross talk between channels, as described previously [14]. Some individual images were acquired using a Nikon E800 fluorescence microscope (Nikon, Japan), by a Coolsnap EZ CCD Camera (Photometrics, Tucson, AZ, USA), and analyzed using MetaMorph image analysis software (Molecular Devices, San Francisco, CA, USA). 

### 2.5. Statistical Analysis

The data of comparative gene expression of the four stem cell transcription factors and the fibroblast markers between the groups and immunocytochemical-positive cells were all recorded and expressed as the mean ± standard deviation. The data among the groups were analyzed by one-way analysis of variance (ANOVA) followed by a Bonferroni post-hoc test (equal variances assumed). A statistical probability of *p* < 0.05 was considered as statistical significance. The software for statistical analysis was IBM SPSS Statistics (Armonk, NY, USA).

## 3. Results

### 3.1. Morphology of the Human NEDAPS Cells

**Figure 1:**
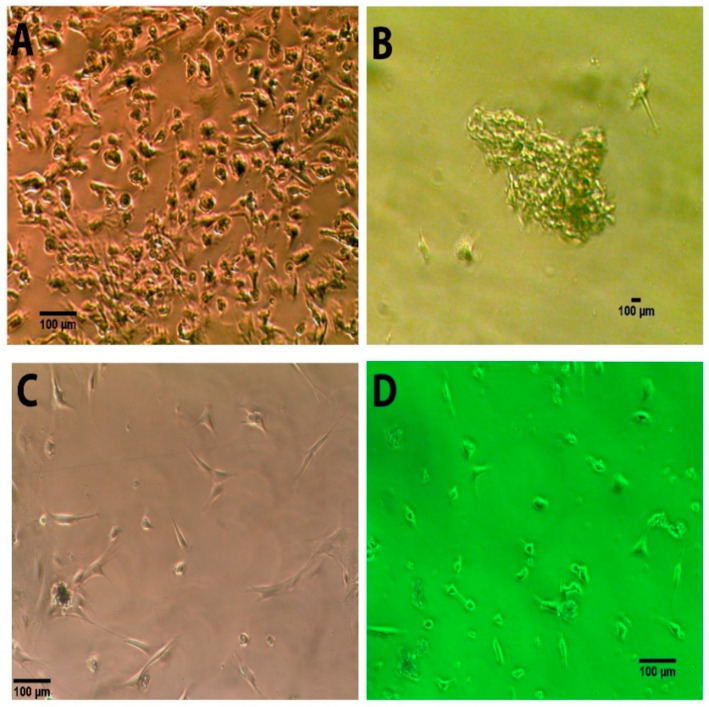
Significantly more cells were isolated from human peripheral nerve tissue pretreated with BMP-2 and propagated in complete stem cell restricted medium at 24 h (**A**) and 7 days (**B**), compared with cells obtained from nerve pieces without BMP-2 pretreatment in complete stem restricted medium at 24 h (**C**) and 7 days (**D**).

Pretreatment of the human nerve pieces with BMP-2 appeared critical in obtaining much more cells for in vitro cultures (Figure 1). Similar to mouse NEDAPS cells, the isolated human cells following BMP-2 treatment readily adhered to the culture plates or chamber-slides and maintained a polygon-shaped morphology (Figure 1A). They also did not require feeder cells to survive. However, in contrast to the rodent cells [14] that remained dispersed on the culture plate, the human cells tended to gather to form colonies. It appears that the motile cells formed clusters within 24 to 48 h in the stem cell medium and stayed in colonies during proliferation. Indeed, the cells in colonies continued to divide and could reach several cell layers (Figure 1B). Maintenance of the cell culture in complete stem cell medium and subcultures every two weeks did not see obvious morphological changes for at least two months.

### 3.2. Expression of Stem Cell Markers

**Figure 2:**
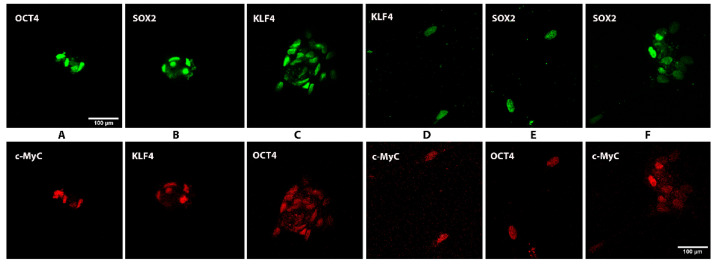
Immunofluorescent staining was performed to identify co-expression of the pluripotent stem cell markers. Cells in wells of a chamber slide were respectively double stained with cross-paired primary antibodies against the specific stem cell markers (**A**–**F**) raised in different species, followed by secondary antibodies probed with Alexa Fluor^®^ 488 (green) and Alexa 594 (red) fluorescent tags.

**Figure 3:**
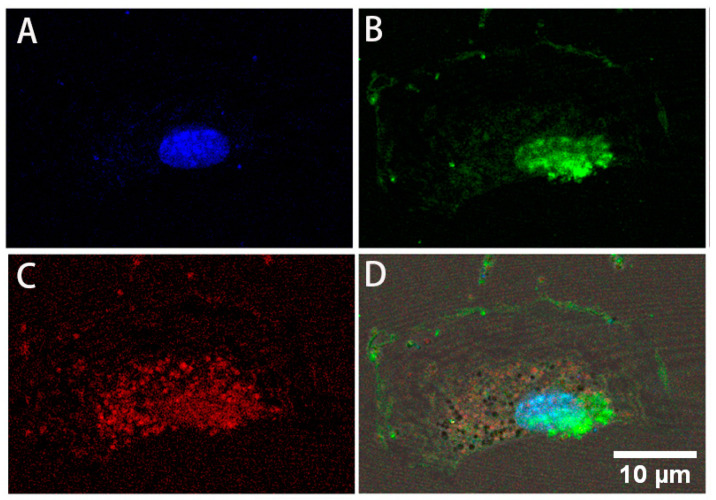
Confocal microscopy images illustrating a human NEDAPS cell: (**A**) DAPI staining the nucleus, (**B**) Sox2, (**C**) Klf4, and (**D**) overlay of the multiple staining (mag. 640×).

Immunofluorescent microscopy on the isolated human nerve cells in complete stem cell medium for at least 7 days clearly showed that over 90% of cells were simultaneously expressing the four transcription factors Sox2, Oct4, c-Myc, and Klf4 after 5 days in culture in the restrictive stem cell medium. Double staining with various pairs of primary antibodies against the transcription factors illustrates that the cells co-expressed all four specific stem cell transcription factors (Figure 2). Figure 3 shows a human NEDAPS cell that was double visualized by Sox2 (green fluorescent) and Klf4 (red fluorescent) under a confocal microscope, with DAPI (blue) for nuclear staining.

**Figure 4:**
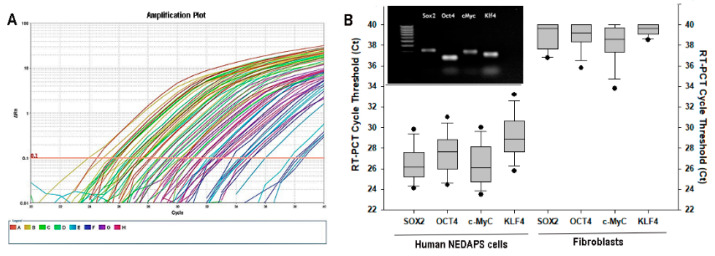
(**A**) An example of a Real-Time PCR amplification plot indicating the replication curves of the specific mRNA tested, with SYBR Green labeling. (**B**) Cycle Thresholds of the four stem cell markers in human NEDAPS cells and the control fibroblastic cells were summarized. The insert in (**B**) illustrates an electrophoresis image of PCR products after the Real-Time PCR, with specific primers for the four transcription factors, on the BMP-2-treated cells isolated from human peripheral nerves. The first lane shows a 100 pb DNA ladder.

Real-Time PCR was performed to examine the mRNA expressions of Sox2, Oct4, c-Myc, and Klf4 of these cells. It is convincing that strong expressions of all the 4 transcription factors were exhibited in the human nerve specimens treated with BMP-2, whereas the non-nerve fibroblastic cells had no expressions (Figure 4). Some of the samples following Real-Time PCR were electrophoresed on an agarose gel to reveal the correct-sized PCR product of the transcription factors (Figure 4B, insert).

### 3.3. Differentiation to Fibroblasts

**Figure 5:**
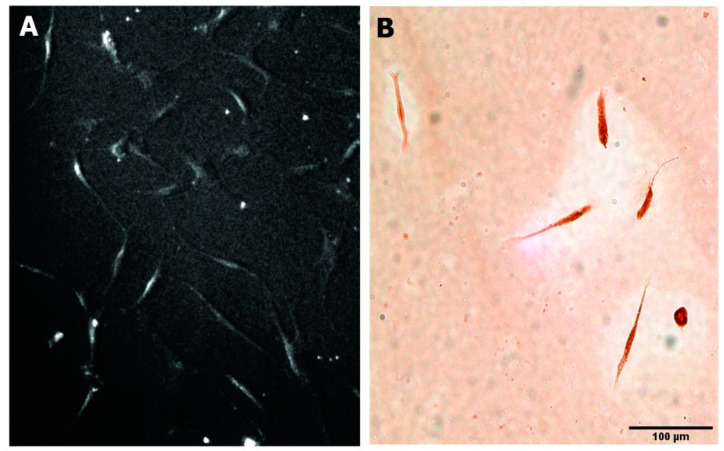
Human NEDAPS cells were morphologically changed to spindle-shaped fibroblastic cells (**A**) and stained strongly positive for fibroblast activation protein alpha positive (**B**).

When the culture media for the human NEDAPS cells were changed to the complete fibroblast induction medium, the cells rapidly dispersed from their small colonies and assumed the classic “spindle shape” of motile fibroblasts within 24–48 h (Figure 5). Immunohistochemical staining against fibroblast activation protein (FAP) confirmed that they become FAP positive.

## 4. Discussion

Bone Morphogenetic Proteins (BMPs), first described by Marshall Urist in 1965 [20], are able to stimulate the stem cell to differentiate into an osteoblast cell, and are considered to be the most important signaling molecules in bone formation and fracture healing. As members of the TGF-β superfamily, the details of their status as intercellular signal transduction are still under study. Among the 20 different human BMPs identified until now, only BMP-2 and BMP-7 are approved by the Food and Drug Administration (FDA) for clinical use. In recent years, more and more orthopedic surgeons are applying BMP-2 to a variety of different clinical applications for its osteoinductive activity [21]. With the increase in clinical use of BMP-2, many associated adverse events have been reported, most of them related to the use of BMP-2 (Infuse) in proximity to nerves [22]. We speculate that the use of BMP-2 in proximity to nerves at supraphysiologic levels can lead to nerve dysfunction as the supraphysiologic induction of stem cell proliferation will result in a likelihood of permanent nerve dysfunction [14]. The data from this study suggested that BMP-2 may effectively stimulate the quiescent population of the pluripotent stem cells to fast proliferation and exhibit their stem-like cell behaviors.

While using a mouse model to investigate the potential complications of BMP-2 to peripheral nerves during bone healing applications, an intriguing finding was noticed: a population of cells was proliferating within the nerve. Indeed, these cells were able to express the four transcription factors c-Myc, Klf4, Sox-2, and Oct-4 [14], suggesting their pluripotent potential. Further investigation also confirmed that physical injury to peripheral nerves, such as compression, also induced the same phenomenon of cell proliferation [14]. In the current study, it is very interesting to note that the same population of cells can be isolated from human peripheral nerve tissue after in vitro incubation with a small amount of BMP-2. Both Real-Time PCR and immunofluorescent staining confirmed that the human NEDAPS cells express the four embryonic stem cells; however, their morphology and motile behavior contrast with the rodent NEDAPS cells that continue to move about on a plastic or glass substrate until the culture surface is confluent with cells. The human cells stop their motile behavior when encountering another NEDAPS cell. Over time, these human NEDAPS cells will form a (non-clonal) colony of cells, due to both additional recruitment of cells as well as ongoing cell division within the colony. This process differs from that of induced pluripotent cells (iPCs) as they are not motile and normally form “clonal” colonies. The potential mechanism is yet to be understood, though. 

Although we have confirmed that rodent NEDAPS can be induced into osteoblastic cell and endothelial cells [13], as well as potentially many others, such as nerve stem cells and Definitive Endoderm cells (unpublished data), the objective of this study is to identify if human NEDAPS cells presented and if they had differentiation potential. To start the exploration, we switched the cell culture medium from a stem cell medium to fibroblast growth medium containing recombinant human Fibroblast Growth Factor-2 (rhFGF-2). The cell morphology was quickly changed within 2–3 days, and IHC staining confirmed expression of fibroblast activation protein (FAP-α). FAP-α has been identified as a highly specific fibroblast marker that is overexpressed in activated fibroblasts or mesenchymal stromal cells [23,24]. We believe that these naturally occurring cells represent the basis of a previously unappreciated universal mechanism for the healing of injured tissues. Ever since the classic work of Dr. Grillo [25], the source of the fibroblasts that have such a prominent role in nearly all mammalian healing events remains unknown. A preliminary animal experimentation of skin wound healing further suggested that NEDAPS cells participate and promote the healing of skin lesions. It postulates that the group of NEDAPS within peripheral nerves based on the results presented here are likely to represent the original cell source of the fibroblasts, and that these nerve-derived cells are necessary for most of the healing events in the body.

We are excited to report on this very recently discovered source of pluripotent cells, which would seem to have an exciting potential for human cell-based therapies. These cells are notably different from embryonic stem cells or the induced pluripotent stem cells (iPCs), with a remarkably distinct appearance and behavior compared to previously described “true embryonic stem cells” [26]. Importantly, these peripheral nerve-derived cells actually seem to have the natural function of accomplishing tissue repair, and their “niche” within peripheral nerves provides a welcome insight into the pathophysiology of the wound-healing problems associated with peripheral neuropathies, including leprosy, diabetes mellitus, and tertiary syphilis [27]. We speculate that the loss of healthy nerves in these patients means that the NEDAPS cells are no longer available to heal such wounds. This insight may open the door to all manners of cell-based treatments using self-to-self autografts of pluripotent NEDAPS cells or differentiated cells therefrom. This could potentially allow for the harvest of non-essential peripheral nerves, such as a branch of the purely sensory sural nerve of the anterior intraosseous nerve (or many others), which could have very wide application for tissue regeneration.

## 5. Conclusions

This preliminary report suggests that the human peripheral nerves also contain normally quiescent NEDAPS cells that exhibit similar but not identical characteristics as rodent NEDAPS cells. Current investigations are focused on further documenting their differentiation potential and to exploring their therapeutic potential in bone and other tissue repair and regeneration models. We suggest that these cells are possibly at the core of a previously unknown natural mechanism for healing an injury.

## 6. Patents

Some of the findings from this research have been filed as part of a patent application (20190218543), “Mammalian pluripotent stem cells, methods for their production and uses thereof”.

## Figures and Tables

**Table 1:** Primers utilized for the RT-PCR amplification of the human specimens.

Target	Forward Primer	Reverse Primer	Product Size
Sox2	agaaccccaagatgcacaac	gggcagcgtgtacttatcct	200
c-Myc	acccgctcaacgacagcagc	ccgtggggaggactcggagg	104
Klf4	ctgaacagcagggactgtca	gtgtgggtggctgttctttt	218
Oct4	agcgatcaagcagcgactat	agagtggtgacggagacagg	202

## Data Availability

Not applicable.
